# Hypovitaminosis D and Leukocytosis to Predict Cardiovascular Abnormalities in Children with Kawasaki Disease: Insights from a Single-Center Retrospective Observational Cohort Study

**DOI:** 10.3390/diagnostics14121228

**Published:** 2024-06-12

**Authors:** Donato Rigante, Gabriella De Rosa, Angelica Bibiana Delogu, Giulia Rotunno, Rossella Cianci, Claudia Di Pangrazio, Giorgio Sodero, Umberto Basile, Marcello Candelli

**Affiliations:** 1 Department of Life Sciences and Public Health, Fondazione Policlinico Universitario A. Gemelli IRCCS, 00168 Rome, Italy; 2 Department of Pediatrics, Università Cattolica Sacro Cuore, 00168 Rome, Italy; 3 Department of Translational Medicine and Surgery, Fondazione Policlinico Universitario A. Gemelli IRCCS, 00168 Rome, Italy; 4 Department of Clinical Pathology, Santa Maria Goretti Hospital, 04100 Latina, Italy; 5 Department of Emergency Anesthesiological and Reanimation Sciences, Fondazione Policlinico Universitario A. Gemelli IRCCS, 00168 Rome, Italy; marcello.candelli@policlinicogemelli.it

**Keywords:** Kawasaki disease, child, coronary artery abnormalities, cardiovascular abnormalities, vitamin D, neutrophil count, innovative biotechnologies

## Abstract

*Introduction:* An aberrant immune response involving yet unidentified environmental and genetic factors plays a crucial role in triggering Kawasaki disease (KD). *Aims:* The aim of this study was to assess general and laboratory data at the onset of KD in a single-center cohort of children managed between 2003 and 2023 and retrospectively evaluate any potential relationship with the development of KD-related cardiovascular abnormalities (CVAs). *Patients and methods:* We took into account a total of 65 consecutive children with KD (42 males, median age: 22 months, age range: 2–88 months) followed at the Department of Life Sciences and Public Health in our University; demographic data, clinical signs, and laboratory variables at disease onset, before IVIG infusion, including C-reactive protein, hemoglobin, white blood cell (WBC) count, neutrophil count, platelet count, aminotransferases, natremia, albumin, total bilirubin, and 25-hydroxyvitamin D were evaluated. *Results:* Twenty-one children (32.3% of the whole cohort) were found to have echocardiographic evidence of CVAs. Univariate analysis showed that diagnosis of KD at <1 year or >5 years was associated with CVAs (*p* = 0.001 and *p* = 0.01, respectively); patients with CVAs had a longer fever duration and mostly presented atypical or incomplete presentations. Interestingly, all patients with CVAs had lower levels of vitamin D (less than 30 mg/dL, *p* = 0.0001) and both higher WBC and higher neutrophil counts than those without CVAs (*p* = 0.0001 and *p* = 0.01, respectively). Moreover, blood levels of albumin were significantly lower in KD patients with CVAs compared to those without (11/21, 52% versus 13/44, 30%, *p* = 0.02). Multiple logistic regression with correction for sex showed that serum vitamin D < 30 ng/mL, WBC count > 20.000/mm^3^, and age > 60 months at KD onset were the only independent factors statistically associated with CVAs. *Conclusions:* Hypovitaminosis D, WBC count over 20.000/mm^3^, and age above 5 years at KD onset emerged as independent factors statistically associated with the occurrence of CVAs.

## 1. Introduction

Kawasaki disease (KD), first reported in 1967, is a multisystemic vasculitis that mainly affects infants and young children and represents the leading cause of acquired heart disease in high-income countries, explaining the host of international studies undertaken to elucidate its etiology, expedite diagnosis, and customize selective treatments [1]. Fever persisting for at least 5 days combined with nonspecific clinical signs (i.e., nonpurulent conjunctivitis, oropharyngitis, polymorphous skin eruption, extremity or perineal changes, and cervical lymph node enlargement) characterizes *typical* KD [2]. Vascular inflammation in KD bears a risk of permanent damage to coronary arteries, and such complications may occur in patients who do not receive proper treatment in due time with intravenous immunoglobulins (IVIGs), aspirin, or corticosteroids [3]. In the acute phase of KD, an immune alteration involving autoinflammatory pathways drives a massive pro-inflammatory cytokine cascade that affects endothelial cells. However, the key steps that lead to coronary arteritis in KD remain largely undeciphered [4,5]. Recurrence of fever in KD children who have been treated with IVIGs is a challenge for pediatricians, who need to be able to predict at disease onset those patients who are at higher risk of developing coronary artery or any cardiovascular abnormalities (CVAs) in the mid-term. The main goal of this study was to evaluate general, clinical, and laboratory parameters of a single-center cohort of children with KD and, in particular, to retrospectively define if any clues at disease onset could eventually predict the occurrence of CVAs.

## 2. Patients and Methods

### 2.1. Study Population

We analyzed retrospectively the medical records of patients who were managed for KD in the Department of Life Sciences and Public Health of Università Cattolica Sacro Cuore, Rome, from January 2003 to December 2023: 72 children were initially considered, but 7 were excluded from our analysis because of missing information in their medical charts. The final number of children with KD was 65: 42 males and 23 females, median age: 22 months, age range: 2–88 months, interquartile range (IQR): 11–45 months; 3 patients were Latin Americans, 2 were Eastern Asians, and the other 60 were of Caucasian ancestry. Diagnosis of KD was established according to the 2006 EULAR/PReS criteria [6]. No patient presented any laboratory signs of macrophage activation syndrome [7], and no patient had a previous history of SARS-CoV-2 infection from 2020 onwards.

All the patients’ parents or guardians were informed about the goal of this retrospective study at a routine outpatient assessment, and all signed a written informed consent for both unrestricted access to medical records and evaluation of their children’s anonymized data. The whole retrospective study was conducted in accordance with the 1996 Helsinki Declaration. The local Ethics Committee authorized a series of study protocols related to nutritional and environmental issues in patients with complex diseases, such as hereditary disorders and autoinflammatory or rheumatologic diseases (approval code: 2105; approval date: 5 February 2019). All recruited children lived in central Italy (latitudes varying from 41°13′ N to 42°42′ N; ultraviolet index: 1–5 during wintertime). Periods at which KD occurred varied seasonally: 38 patients were managed during fall/winter (58%), and 27 were managed during spring/summer (42%). In the whole cohort, 18 (27%) had an *incomplete* form of KD, while 18 (27%) had *atypical* KD, requiring a thoughtful differentiation from autoinflammatory syndromes, which are rare childhood-onset disorders of innate immunity characterized by complex patterns of fever and disease-specific patterns of aseptic organ inflammation involving skin, joints, serosal surfaces, the central nervous system, or other tissues [8].

All our patients received the “standard” treatment with IVIGs (2 g/kg of body weight) before the 10th day of illness after fever onset and aspirin (30–50 mg/kg daily, divided into four doses, orally); after defervescence, aspirin was switched to one single daily dose of 3–5 mg/kg of body weight for at least 8 weeks or for different periods as judged by cardiologic assessment on a case-by-case basis. IVIG treatment was well tolerated, and no patients developed side effects. Nonresponsive patients were given second-line treatments for KD, as suggested by current guidelines: treatment of patients failing to respond to IVIGs is not fully standardized, but a second IVIG cycle, followed by second-line therapies, including methylprednisolone and biologics, are recommended in these cases [9]. Patients were followed up for different periods, with the oldest cases being evaluated at least once per year after illness. Results of echocardiography during the KD acute phase were available for all patients. All outcomes related to cardiovascular-related mortality, eventual *all*-cause hospital admissions, cardiovascular-related hospital admissions, and general health issues were assessed for all cases.

### 2.2. Data Collection

For each patient, demographic data (date of birth, sex, skin color, ethnicity, age at KD onset), clinical signs suggesting KD (including the days between disease onset and the start of the treatment), and laboratory variables at the onset of KD were evaluated: C-reactive protein [CRP], hemoglobin [Hb], white blood cell [WBC] count, neutrophil count, platelet count, alanine aminotransferase [GOT], aspartate aminotransferase [GPT], natremia, albumin, total bilirubin, and 25-hydroxyvitamin D [25(OH)-vitamin D]. Sampling was performed before IVIG infusion as well as before introducing aspirin. Serum 25(OH)-vitamin D levels were measured by automated chemiluminescence immunoassay technology. Vitamin D values were categorized as being “sufficient” when over 30 ng/mL, “insufficient” when between 20 and 30 ng/mL, and “deficient” when less than 20 ng/mL, based on the reference values published by Holick in 2007 [10]. N-terminal pro-B-type natriuretic peptide (n.v. < 158 pg/mL) was only assessed in a minority of KD patients of the cohort, from 2015 onwards, and therefore it was disregarded in the statistical analysis. No cases of bacteremia were found in these children.

Each patient was also evaluated by the pediatric cardiologist through electrocardiography and 2D echocardiography at diagnosis (within 10 days after fever onset and always before IVIG administration), then at 1, 2, 3, and 4 weeks, and with monthly frequency during the first six months after KD diagnosis to assess any CVAs. Coronary artery dilation was defined as a coronary diameter larger than 3 mm in children under 5 years and larger than 4 mm if older; coronary artery involvement (dilatation as well as small, medium, or large aneurysms) was determined by body surface area-based *z*-scores, using the *z*-score stratification proposed by the American Heart Association guidelines: no involvement for a *z*-score < 2, dilation for a *z*-score between 2 and 2.5, small aneurysm for a *z*-score ≥2.5 and <5, medium aneurysm for a *z*-score ≥5 and <10, and large or giant aneurysm for a *z* score ≥ 10 [11]. When *z*-scores could not be calculated because of missing details about coronary artery diameters, the degree of involvement was based on the specific cardiologist’s report. Further cardiovascular complications, including myocarditis and valvular regurgitations, were assessed during the acute phase, and follow-up was completed at different intervals depending on the individual patient’s needs. Computed tomography coronary angiography was performed in selected patients who had large or unusual coronary artery lesions or if their visualization was difficult. We periodically assessed all the outcomes related to hospital admissions or eventual KD-related complications requiring hospitalization for every patient. Periodic complete heart assessments were ensured for all consecutive KD children, who were spread throughout the 2003–2023 period: overall, patient follow-up was completed for a mean period of at least 7 years via yearly echocardiography, and most patients are still looked after. No child with KD died in our cohort during the observation period.

### 2.3. Statistical Analysis

We used the IBM SPSS software version 22 for statistical analysis. Continuous data were presented as means and standard deviations (SDs) for normally distributed variables and as medians and IQRs for non-normally distributed variables. Categorical data were described using frequencies and percentages. The Student’s *t*-test and the Mann–Whitney U test were employed for comparing normally and non-normally distributed groups, respectively. The chi-square test and Fisher’s exact test were used for comparing categorical data between the groups. To include continuous variables in the multivariate logistic regression analysis, we constructed a receiver operating characteristic (ROC) curve to calculate the accuracy of diagnosing CVAs and the optimal cut-off value (using the Youden index). These curves were generated only for continuous variables with a *p*-value < 0.15 in the univariate analysis. Following this, we categorized these variables based on values above or below the calculated cut-off. We then performed the chi-square test to evaluate significant differences in the newly categorized variables between patients with and without CVAs. Finally, all variables with a *p*-value < 0.15 in the univariate analysis were incorporated into a multiple logistic regression model, adjusted for sex, to assess independent associations with CVAs in our patients. A *p*-value < 0.05 was considered statistically significant.

## 3. General Results

### 3.1. Clinical Features of Children with KD and Their Responses to Treatment

The demographic and KD-specific characteristics of all the participants are shown in Table 1. Fourteen out of sixty-five patients (22%) were nonresponsive to the first IVIG infusion (given at the standard dose) and required a second infusion afterward. Six out of sixty-five patients (9% of the whole cohort), still refractory after two cycles with IVIGs, were treated with methylprednisolone pulses (30 mg/kg/day for three days); two patients received the chimeric monoclonal anti-tumor necrosis factor antibody drug infliximab (5 mg/kg of body weight in a single intravenous infusion); and two received the recombinant interleukin-1 receptor antagonist anakinra (6 mg/kg of body weight per day by subcutaneous injection for a variable period depending on the specific cardiovascular assessments). No secondary effects occurred during anakinra treatment, and discontinuation of anakinra was not followed by any inflammatory relapses in the two cases.

Among the patients, 21 (32.3% of the cohort, with a median age of 11 months, an age range of 2–88 months, and an IQR of 7–61 months) were found to have echocardiographic evidence of CVAs in terms of perivascular brightness of coronary arteries and changes in coronary artery diameters, which were collectively considered. The sites of coronary artery lesions were comparable to those found in other studies: the left anterior descending artery, the left main coronary artery, and the right coronary artery. Giant coronary artery aneurysms, confirmed by computerized tomography coronary angiography, occurred in only one case and involved the left anterior descending artery (*z*-score: 11.2) and the right coronary artery (*z*-score: 11.4). Among the patients with non-aneurysmatic cardiovascular lesions, two patients had myocarditis with myocardial dysfunction, four had mitral valve insufficiency, and one had aortic insufficiency. No major adverse cardiac events were reported by patients during the follow-up.

We classified patients into two groups according to whether they had CVAs (in different forms) or not (see Table 1). Silent myocardial ischemia was not observed, nor were ischemic changes found in routine electrocardiograms in the cardiologic assessments. KD-related coronary artery lesions, including both giant coronary artery aneurysms and signs of valvular involvement, disappeared after a variable period during the follow-up in all cases.

### 3.2. Univariate Analysis

**Figure 1:**
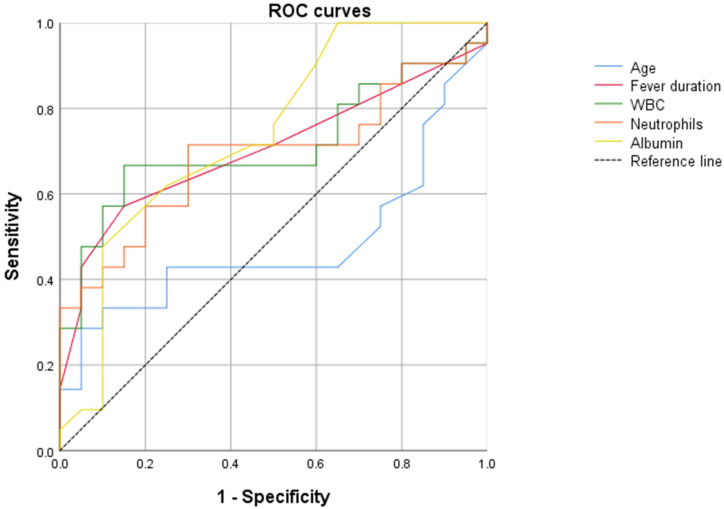
ROC curve analysis related to age, fever duration, white blood cell count, neutrophil count, and albumin for diagnosing cardiovascular abnormalities in patients with Kawasaki disease. ROC: receiver operating characteristic, WBC: white blood cell.

In the univariate analysis, we found that diagnosis of KD at an age <1 year or >5 years was associated with the development of CVAs (*p* = 0.001 and *p* = 0.01, respectively). Patients with CVAs had a longer fever duration and showed a tendency to present atypical or incomplete disease patterns. Interestingly, all patients with CVAs had lower values of vitamin D (less than 30 mg/dL, *p* = 0.0001) and both higher WBC and higher neutrophil counts than those without CVAs (*p* = 0.0001 and *p* = 0.01, respectively). Moreover, blood levels of albumin were significantly lower in KD patients with CVAs compared to those without (11/21 (52%) versus 13/44 (30%), *p* = 0.02). To include continuous variables in our multiple logistic regression model, we performed an ROC curve analysis for all continuous variables with *p* < 0.15 in the univariate analysis; then, we evaluated the diagnostic accuracy by means of the area under the ROC (AUROC) and the best cut-off through Youden’s index (Figure 1, Table 2).

Subsequently, we used these cut-offs to transform continuous data into categorical dichotomic data (above or under the cut-off value) and applied the chi-square test or Fisher’s exact test to compare KD patients with and without CVAs (see Table 3).

### 3.3. Multivariate Analysis

**Figure 2:**
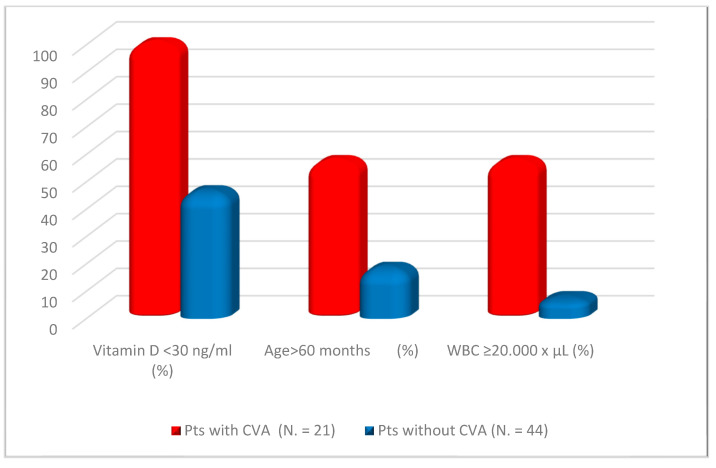
Factors independently associated with the occurrence of cardiovascular abnormalities (CVAs) at disease onset in our cohort of children with Kawasaki disease: hypovitaminosis D, age over 60 months (5 years), and white blood cell count over 20.000/mm^3^. CVAs: cardiovascular abnormalities, WBC: white blood cell, No.: number.

We performed a multivariate analysis including all variables, with *p* < 0.15 in the univariate evaluation and correction for patient sex (Table 4). Levels of 25-OH-vitamin D < 30 ng/mL, WBC counts > 20.000/mm^3^, and age > 60 months at KD onset were the only independent factors statistically associated with the occurrence of CVAs (Figure 2).

## 4. Discussion

The worldwide spread of KD and the crucial role of early diagnosis in preventing CVAs have created the need for periodic updates of guidelines dedicated to KD management. These updates should aim to refine the understanding of the disease and implement rigorous assessments of treatment efficacy. Traditionally, the lack of defervescence within 24–36 h in children with an established diagnosis of KD after IVIG administration or the recurrence of fever after an initial response have been considered cues to recommend further second-line treatments [9,12]. Many attempts to develop universal scoring systems and detect children at higher risk of IVIG resistance have been unsuccessful, and KD may show a different evolution according to yet unraveled demographic, genetic, or epigenetic factors [13]. A network of genes orchestrating inflammatory machineries might be involved in the pathogenesis, development, and evolution of KD: several studies on gene expression and genome-wide associations in patients with KD have found that the most likely potential susceptibility genes are *ITPKC*, *CASP3*, *FCGR2A*, and *KCNN2* and have also confirmed that innate immunity pathways related to pathogen-associated molecular patterns of infectious agents and extracellular matrix components and proteins regulating their remodeling might be involved [14].

About 10–20% of patients with KD are nonresponsive to IVIGs, requiring secondary treatments, and may display an increased risk of CVAs: their early identification is critical to reinforce the standard initial approach to such patients, but all available scoring systems are unsuitable in non-Japanese populations [15]. We previously found that higher CRP values and younger age at KD onset were associated, respectively, with a failure in IVIG response and a higher risk of CVAs [16]. Additionally, the exact risk of developing cardiovascular events after KD remains undefined, warranting preventive counseling and regular cardiovascular surveillance in all children with a history of previous KD, aiming to mitigate the risk of eventual adult-onset cardiovascular diseases [17].

Our present retrospective single-center cohort study was conducted in the Department of Life Sciences and Public Health of Università Cattolica Sacro Cuore in Rome, Italy: the medical records of 65 consecutive patients (42 males, median age: 22 months, age range: 2–88 months, IQR: 11–45 months) managed for KD from January 2003 to December 2023 were evaluated. The patients’ demographic data, clinical signs evocative of KD, and laboratory variables at onset were assessed and compared with findings related to CVAs as reported in the medical charts. All patients received treatment with IVIGs; 22% received a second cycle with IVIGs (28% of those with CVAs, 18% of those without CVAs); refractoriness to IVIGs related to KD nonresponsiveness, exhibited by persistent or recrudescent fever after the completion of IVIG infusion, requiring secondary treatments, was observed in 9% of patients (19% of those with CVAs, 5% of those without CVAs). Univariate analysis showed that diagnosis of KD at <1 year or >5 years was associated with CVAs and that patients with CVAs had a longer fever duration with frequent atypical or incomplete phenotypes. Moreover, all patients with CVAs had lower levels of vitamin D (less than 30 mg/dL) and higher WBC and neutrophil counts than those without CVAs. Albumin levels were also found to be significantly lower in KD patients with CVAs compared to those without. Multiple logistic regression with correction for sex showed that vitamin D < 30 ng/mL, WBC count > 20.000/mm^3^, and patient age > 60 months at disease onset were the only independent factors statistically associated with CVAs.

The role of vitamin D in the pathogenesis of different inflammatory disorders has been increasingly studied in recent years, but its exact contribution to KD-related vascular abnormalities remains undeciphered. There is mounting evidence that vitamin D deficiency may be associated with the severity of various immune-mediated and inflammatory disorders: different in vitro studies have shown that vitamin D modulates T cell proliferation and dendritic cell function, enhancing T-reg production, downregulating a host of pro-inflammatory cytokines, upregulating anti-inflammatory cytokines, and inhibiting both proliferation and differentiation of B cells [18,19]. Additionally, KD has been reported to occur in 4.7% of patients with periodic fever, aphthous stomatitis, pharyngitis, and cervical adenitis (PFAPA) syndrome, a typical childhood febrile disorder, which is much higher than the incidence of KD (0.02%) reported among children younger than 5 years in San Diego County, USA [20]. Deficient serum vitamin D levels have been found in children with PFAPA syndrome, suggesting this deficiency to have a role in the pathogenesis of febrile flares [21]. On the other hand, epidemiological studies related to KD have indicated an inverse association between vitamin D deficiency, prevalence of CVAs, and further cardiometabolic risk factors such as hypertension, diabetes, dyslipidemia, metabolic syndrome, and metabolic dysfunction-associated steatotic fatty liver disease [22]. In particular, some authors found low vitamin D levels in almost the totality of children with KD compared with healthy controls, and a significantly higher percentage of those who developed CVAs had the lowest vitamin D levels [23]. Furthermore, Suzuki et al. found that adjunctive vitamin D administration may modulate the overall inflammatory response of children with KD [24].

In his landmark paper reporting the first 50 cases of KD, Dr. Kawasaki noted that an elevated number of white blood cells was the striking feature of these patients, and indeed an increased WBC count is considered a marker of active KD and is also suggested as a risk factor for cardiovascular morbidity: the morphologic analysis of neutrophils might even represent a wieldy tool to predict KD severity and poorer response to IVIGs [25]. Additionally, the interleukin-17 A/interleukin-17 receptor A axis has a position in mediating neutrophil chemoattraction in affected vessels before IVIG treatment, thus contributing to the severity of CVAs in children with KD [26]. Another potential association linking white blood cells to CVAs stems from the production of semaphorin 4D by polymorphonuclear cells; indeed, the receptors for this molecule have been identified on endothelial cells and exhibit pro-inflammatory properties. Elevated levels of semaphorin 4D have been observed in KD patients, particularly those with CVAs, and have been correlated with increased levels of metalloproteinases and different pro-inflammatory cytokines [27]. Inter alia, patients with KD, especially those who do not respond to conventional therapies and tend to develop CVAs, show overexpression of CD177 on neutrophil cell membranes: CD177 is a marker of both neutrophil activation and degranulation and appears to be specifically associated with KD, as it significantly decreases after treatment with IVIGs [28,29].

It is also well established that children under 5 years are at higher risk of developing KD compared with other populations, with only 13.2% of all KD cases being older than 5 years [30]. Notably, children over 5 years of age have been reported to have a substantially higher risk of exhibiting CVAs due to delay in diagnosis, as the treating physicians may more frequently consider febrile exanthemata or viral infections instead of KD during differential diagnosis of an older child with persistently unmanageable fever [31]. A nomogram model using the neutrophil-to-lymphocyte ratio is in progress to assess and predict any potential risk of CVAs in older children and adolescents with KD [32].

To determine the disease outcome and risk factors of heart damage, we started this retrospective study evaluating the medical records of all patients with a diagnosis of KD in our department between 2003 and 2023. Among the 65 patients recruited, 21 had CVAs (32.3%) in the form of perivascular brightness and dilatation of coronary arteries, as well as aneurysms combined with myocarditis (in two cases), mitral valve insufficiency (in four cases), and aortic insufficiency (in one case), but all of them displayed a complete regression at different times during the varying periods of follow-up. Although caution is warranted in extrapolating from our results, our study ultimately reveals that the mid- to long-term prognosis of KD that is treated canonically may be excellent and that the majority of affected children do not have any cardiac sequelae and do not suffer from complications in the long term. Univariate analysis showed that diagnosis of KD in younger and older children was associated with CVAs, and, interestingly, all patients with CVAs had insufficient levels of vitamin D and both higher WBC and higher neutrophil counts. The multiple logistic regression with correction for sex showed that insufficient vitamin D, leukocytosis, and older age at disease onset remained independently and significantly related to CVAs in KD.

The present study must be viewed in the light of some limitations. First, it has the shortcomings of all retrospective studies and focused on our single-center cohort observations during a long time frame, which may inherently have introduced selection biases among the patients. Second, the study’s restricted patient population, stemming from the low incidence of KD in Western countries, hindered the exploration of potential correlations among vitamin D levels, leukocytosis, and other markers of disease severity with a lack of response to IVIGs and potential mortality. Third, the guidelines for KD, and therefore for the management of patients in our department, changed throughout the study period, which made the follow-up of all examined patients not uniform and standardized. Of course, it was not possible to perform other comparisons with mortality rates or further outcome measures due to the favorable evolution of KD (no patients died during the long investigation period of follow-up) and due to the low number of nonresponders to IVIGs.

## 5. Conclusions

In conclusion, this study provides a static snapshot of different general and laboratory data at the onset of KD, revealing that hypovitaminosis D, WBC count over 20.000/mm^3^, and older patient age at KD onset might act as independent factors statistically associated with the occurrence of CVAs. Future larger studies are needed to unravel the exact relationship between the protean contributors to KD and different extents of cardiac damage.

## Figures and Tables

**Table 1:** Demographic and specific characteristics of children with Kawasaki disease (KD) assessed in our study in relation to the presence of cardiovascular abnormalities (CVAs) or not.

	All Pts	Pts with CVAs	Pts without CVAs	*p*
No. of KD pts	65	21	44	
Median age in months (age range; IQR)	22 (2–88; 11–45)	11 (2–88; 7–61)	23 (3–73; 14–42)	0.39
No. of male pts (%)	42 (65)	14 (67)	28 (64)	0.81
No. of KD cases in fall/winter (%)	38 (59)	9 (43)	29 (66)	0.08
No. of pts with extremity changes (%)	33 (51)	9 (43)	24 (55)	0.38
No. of pts with mucositis (%)	56 (86)	18 (86)	38 (86)	1.00
No. of pts with cervical adenitis (%)	44 (68)	13 (62)	31 (70)	0.49
No. of pts with conjunctivitis (%)	54 (83)	17 (81)	37 (84)	0.74
No. of pts with polymorphous rash (%)	56 (86)	18 (86)	38 (86)	1.00
No. of complete KD cases (%)	47 (72)	13 (62)	34 (77)	0.2
No. of incomplete KD cases (%)	18 (28)	9 (43)	9 (20)	0.06
No. of atypical KD cases (%)	18 (28)	9 (43)	9 (20)	0.06
Median of days of fever duration (IQR)	8 (7–8)	8 (6–10)	6.5 (5,75–8)	0.02
No. of pts who received a second IVIG cycle (%)	14 (22)	6 (28)	8 (18)	0.34
C-reactive protein, in mg/L (m ± SD)	130 ± 78	149 ± 89	121 ± 72	0.22
Hemoglobin, in g/dl (m ± SD)	10.7 ± 1.1	10.4 ± 1.3	10.8 ± 0.9	0.20
WBC count, in mm^3^ (m ± SD)	17,066 ± 7615	21,869 ± 10,391	14,774 ± 4412	0.006
Neutrophil count, in mm^3^ (m ± SD)	12,091 ± 6992	16,073 ± 9980	10,190 ± 3873	0.016
Platelet count, in mm^3^ (m ± SD)	435 ± 173	462 ± 235	423 ± 136	0.49
No. of pts with platelet counts < 300.000/mm^3^ (%)	9 (14)	5 (24)	4 (9)	0.13
GOT (m ± SD)	67 ± 112	63 ± 111	69 ± 114	0.85
GPT (m ± SD)	81 ± 111	73 ± 117	85 ± 109	0.70
Sodium, in mEq/L (m ± SD)	134 ± 3.4	134 ± 2.9	134 ± 3.6	0.65
Albumin, in g/dL (m ± SD)	3.5 ± 0.6	3.2 ± 0.6	3.6 ± 0.5	0.01
Median of total bilirubin, in ng/mL (IQR)	0.7 (0.5–0.8)	06 (0.5–0.8)	0.7 (0.6–0.825)	0.60
25(OH)-vitamin D, in ng/mL (m ± SD)	26.9 ± 10.3	19.8 ± 4.7	30.2 ± 10.6	0.0001
No. of pts with 25(OH)-vitamin D < 20 ng/mL (%)	24 (37)	12 (57)	12 (27)	0.02
No. of pts with 25(OH)-vitamin D in the range of 20–30 ng/mL (%)	17 (26)	9 (43)	8 (18)	0.03
No. of pts with 25(OH)-vitamin D < 30 ng/mL (%)	41 (63)	21 (100)	20 (46)	0.0001

Pts: patients, No.: number, IQR: interquartile range, IVIG: intravenous immunoglobulin, m: mean, SD: standard deviation, GOT: alanine aminotransferase, GPT: aspartate aminotransferase, WBC: white blood cell.

**Table 2:** Accuracy and best cut-off values for diagnosing cardiovascular abnormalities in patients with Kawasaki disease in relation to age, fever duration, white blood cell count, neutrophil count, and serum albumin evaluated using AUROC curves and Youden’s index.

	AUROC	*p*	Best Cut-Off
Age	0.452	0.543	60 (months)
Fever duration	0.668	0.033	9 (days)
WBC count	0.751	0.001	20,000 (mm^3^)
Neutrophil count	0.724	0.004	11,500 (mm^3^)
Albumin	0.693	0.014	4 (g/dL)

AUROC: area under the receiver operating characteristic, WBC: white blood cell.

**Table 3:** Univariate analysis to compare patients with Kawasaki disease who had cardiovascular abnormalities (CVAs) and those who did not after transformation of continuous variables into dichotomic variables (above or under the best cut-off value).

	All Patients	Pts with CVAs	Pts without CVAs	*p*
No. of pts with age ≥ 60 months (%)	20 (31)	12 (57)	8 (18)	0.001
No. of pts with fever duration ≥ 9 days (%)	11 (17)	8 (38)	3 (7)	0.003
No. of pts with WBC count ≥ 20.000/mm^3^ (%)	15 (23)	12 (57)	3 (7)	0.0001
No. of pts with neutrophil count ≥ 11.500/mm^3^ (%)	28 (43)	14 (67)	14 (32)	0.01
No. of pts with albumin ≥ 4 g/dL (%)	7 (18)	2 (9)	5 (9)	1

No.: number, pts: patients; WBC: white blood cell.

**Table 4:** Multivariate logistic regression analysis to identify factors associated with the occurrence of cardiovascular abnormalities (CVAs) in patients with Kawasaki disease (KD).

	Pts with CVAs (No. = 21)	Pts without CVAs (No. = 44)	*p*	*OR (95%CI)*
No. of pts with age ≥ 60 months (%)	12 (57)	8 (18)	0.037	11.80 (1.335–19.223)
No. of male pts (%)	42 (65)	14 (67)	0.292	0.225 (0.014–3.591)
No. of KD cases in fall/winter (%)	9 (43)	29 (66)	0.378	4.079 (0.028–5.052)
No. of incomplete KD cases (%)	9 (43)	9 (20)	0.060	32.356 (0.865–121.214)
No. of atypical KD cases (%)	9 (43)	9 (20)	0.135	12.814 (0.454–36.944)
No. of pts with 25(OH)-vitamin D < 30 ng/mL (%)	21 (100)	20 (46)	0.033	36.356 (1.329–98.892)
No. of pts with WBC ≥ 20.000/mm^3^ (%)	12 (57)	3 (7)	0.016	49.303 (4.765–508.672)
No. of pts with neutrophils ≥ 11.500/mm^3^ (%)	14 (67)	14 (32)	0.166	0.016 (0.589–5.604)
No. of pts with platelet count ≤ 300.000/mm^3^ (%)	5 (24)	4 (9)	0.900	1.236 (0.046–22.254)

No.: number, pts: patients; WBC: white blood cell.

## Data Availability

The original contributions presented in the study are included in the article, further inquiries can be directed to the corresponding author.
